# Effects of acute hemorrhage on intrapulmonary shunt in a pig model of acute respiratory distress-like syndrome

**DOI:** 10.1186/s12890-016-0221-5

**Published:** 2016-04-26

**Authors:** Nils Siegenthaler, Raphael Giraud, Delphine S. Courvoisier, Claes U. Wiklund, Karim Bendjelid

**Affiliations:** Intensive Care Unit, University Hospitals of Geneva, Geneva, Switzerland; University of Geneva, University Medical Center, Geneva, Switzerland; Division of Clinical Epidemiology (Biostatistics), University Hospitals of Geneva, Geneva, Switzerland; Department of Physiology and Pharmacology, Division of Anaesthesiology and Intensive Care, Karolinska Institute, Stockholm, Sweden; Geneva Hemodynamic Research Group, Geneva, Switzerland; Department of Anesthesiology, Pharmacology and Intensive Care, Service of Adult Intensive Care, Geneva University Hospital, Rue Gabrielle-Perret-Gentil 4, 1205 Genève, Switzerland

**Keywords:** Transpulmonary blood flow, Lung compliance, Admission

## Abstract

**Background:**

In acute respiratory distress syndrome (ARDS), gas exchange and respiratory system mechanics (compliance) are severely impaired. Besides ventilatory parameters, the degree of respiratory abnormality can be influenced by the circulatory state. This study investigated the influence of acute hypovolemia on the respiratory system.

**Methods:**

We performed a secondary analysis of a previous study including 8 pigs with ARDS-like syndrome induced by lung lavage and surfactant depletion method (ARDS group) and 10 mechanically ventilated pigs with no intervention (CTRL group). Animals of both groups were subjected to hemorrhage and retransfusion successively. We reanalyzed the effect of acute blood volume variations on intrapulmonary shunt (shunt), arterial oxygenation (PaO_2_:FiO_2_), global oxygen delivery (DO_2_) and respiratory system compliance (Crs).

**Results:**

In the ARDS group, after hemorrhage, shunt decreased (−28 +/− 3.5 % (*p* < 0.001)), respiratory system compliance (Crs) increased (+5.1 +/− 1.0 ml/cm H2O (*p* < 0.001)) moreover, there was a concurrent increase in PaO2:FiO2 (+113 +/− 19.1 mmHg; *p* < 0.001) but this did not prevent a reduction in DO_2_ (−317 +/− 49.8 ml/min; *p* < 0.001). Following retransfusion, shunt and Crs return towards pre-hemorrhage values. Similar changes, but of smaller magnitude were observed in the CTRL group, except that no significant changes in oxygenation occurred.

**Conclusions:**

The present analysis suggests that an acute decrease in blood volume results in a decrease in shunt with a parallel improvement in arterial oxygenation and an increase in Crs during ARDS-like syndrome. Our results strengthen the importance to integrate the circulatory condition in the analysis of the state of the respiratory system. However, the translation of this physiological model in a clinical perspective is not straightforward because our model of acute and severe hemorrhage is not strictly equivalent to a progressive hypovolemia, as could be obtained in ICU by diuretic. Furthermore, the present model does not consider the impact of blood loss induced decrease of DO_2_ on other vital organs function.

**Trial registration:**

‘Not applicable’.

## Background

Several studies explored the influence of ventilation parameters during acute respiratory distress syndrome (ARDS) [1, 2]. However, the influence of hemodynamic parameters on respiratory characteristics is frequently neglected. Indeed, animal studies reported that a decrease in cardiac output (CO), induced by a reduction in blood volume [3–5] or by pharmacological intervention [6, 7], may be associated with a decrease in intrapulmonary shunt, in normal lung [3, 5] as well as during ARDS [3, 4, 6, 7]. In addition, other studies reported an increase in intrapulmonary shunt associated with an increase in CO [5, 8]. Human studies reported similar results, where a parallel change in CO and shunt has been observed in normal subjects during exercise [9] or during CO reduction following the use of positive end-expiratory pressure (PEEP) in patients with ARDS [10], even if the effect of PEEP on shunt is not only related to his effect on CO [11]. Concerning the respiratory system compliance (Crs), previous studies reported conflicting results as some observed an increase [12, 13], a decrease [5] or no significant changes [14, 15] in Crs related to hemorrhage. On the other hand, an increase in pulmonary blood volume has been shown to result in a decrease in lung compliance [16].

In a previous study exploring the influence of tidal volume (V_T_) and respiratory rate (RR) on pulse pressure variations in pigs with ARDS [17], we observed an unexpected improvement in oxygenation and an increase in Crs during acute hemorrhage. In order to complete previous studies on this issue, we analyzed retrospectively our data with the hypothesis that a decrease in CO due to hypovolemia, may decrease intrapulmonary shunt, improve Crs and may result in an improvement in arterial oxygenation during ARDS.

## Methods

The present study was a second analysis from a previous study [17]. The initial experimentation was performed on 24 domestic pigs. Domestic pigs were selected for the experiment because of their strong similarity in term of physiologic values, hemodynamic and respiratory behavior with humans. In this first analysis only 16 animals have been included as 8 pigs were excluded. Indeed, five animals allowed to improve the model (among them 2 animals died during the experiment), and three animals were excluded because of technical problems (dysfunction of the measurement systems). For the present analysis, 18 animals (mean weight of 31.4 ± 3.1 kg) from the 24 considered in the first study have been included. Four pigs were excluded due to lack of data needed for the present analysis and two other animals died during the experiment.

### Ethics statement

The study was approved by the Ethics Committee for Animal Research at the University Medical Centre and by the Cantonal Veterinary Office of Geneva, Switzerland (No 31.1.1043/3127/1). Animals received respectful care in accordance with the Guide for the Care and Use of Laboratory Animals (Institute of Laboratory Animal Resources, 1996).

### Preparation

Animals were premedicated with intramuscular azaperon 6 mg kg^−1^, midazolam 0.5 mg.kg^−1^, and atropine 0.5 mg. Anesthesia was induced by isoflurane and maintained by fentanyl 20 μg.kg^−1^.h^−1^, isoflurane 1.5–2.0 % and pancuronium 0.4 mg.kg^−1^.h^−1^. Animals were intubated and mechanically ventilated (Servo Ventilator 900, Siemens-Elema, Sweden). FiO_2_ was set at 0.4 for the control (CTRL) group and 1.0 for the ARDS group. Tidal volume (V_T_) was set at 10 ml.kg^−1^ without positive end expiratory pressure (PEEP) and respiratory rate (RR) was 15/min with a fixed inspiratory to expiratory time ratio (1:2). A Swan-Ganz catheter (CCOmboV, 7.5 F, Edwards Lifesciences™, Irvine, CA) was placed in the pulmonary artery. Arterial pressure was recorded in the aorta trough a carotid arterial catheter. A right internal jugular vein catheter was placed for central venous pressure (CVP) measurement, drug infusion and blood retransfusion. Heart rate and rhythm were recorded with a standard 3-lead electrocardiogram.

### Measurements

#### Respiratory system compliance

Airway pressure was monitored (UltimaTM, Datex/Instrumentarium, Helsinki, Finland). V_T_ was calculated by digital integration of a flow signal measured by a pneumotachograph (Gould Godart, model 17212). Quasi-static Crs was calculated after an inspiratory pause of 3 s as V_T_/(plateau airway pressure – PEEP). Flow tracings were visually checked to ensure the absence of signs of intrinsic PEEP [18].

#### Blood gas tensions

Arterial blood gas (ABG) tensions, hemoglobin (Hb), oxygen saturation (arterial blood oxygen saturation (SaO_2_) and mixed venous blood oxygen saturation (SvO_2_) were analyzed by an automated oximeter (ABL-505 Analyzer, Radiometer, Copenhagen, Denmark).

#### Intrapulmonary shunt

In the ARDS group, shunt was calculated using the standard formula: Qs/Qt = (CcO_2_ ‐ CaO_2_)/(CcO_2_ ‐ CvO_2_) with the FiO_2_ set at 1.0.

In the present equation, Qs represents the shunt flow; Qt represents the systemic blood flow, the CcO_2_ represents the pulmonary capillary oxygen content; CaO_2_, the arterial oxygen content and CvO2, the mixed venous oxygen content. Oxygen content in arterial venous and capillary blood where calculated as: (1.34 × oxygen blood saturation × hemoglobin [Hb]) – (PO_2_ × 0.003). In the CTRL group, as FiO_2_ was set at 0.4 therefore, the calculation of shunt represent venous admixture.

#### Global oxygen delivery

DO_2_ was calculated as the product of CaO_2_ and CO, where CaO_2_ was calculated as 1.34 × Hb × SaO_2_ + (0.003 × PaO_2_).

Hemodynamic and respiratory measurements were digitized using an analog/digital interface converter (Biopac, Santa Barbara, CA, USA) and stored for off-line analysis.

### Experimental protocol

Animals were separated into two groups. The CTRL group (*n* = 10) received anesthesia and controlled mechanical ventilation but no intervention. The ARDS group (*n* = 8) was submitted to a procedure of surfactant depletion to induce an ARDS-like syndrome [19]. This procedure consisted of lung lavage with NaCl 0.9 % (1000 ml) at 37 °C repeated until criteria for ARDS were fulfilled (PaO_2_/FiO_2_ < 200 mmHg). This required a mean ± SD of 3.1 ± 0.3 lung lavage at 12.6 ± 3.5 min intervals.

During hemorrhage total blood volume was reduced by 40 % of (estimated at 70 ml.kg^−1^ body weight) over 5–10 min. Removed blood was stored at body temperature in bags containing citrate-phosphate-dextrose (Baxter AG, Volketswil, Switzerland) and was totally retransfused during the retransfusion period. The mean time between the start of bleeding until completed retransfusion was 49 ± 11 min.

Measurement were assessed at pre-hemorrhage, hemorrhage, and retransfusion period in the CTRL group and at baseline (pre-ARDS), pre-hemorrhage (post-ARDS), hemorrhage and retransfusion in the ARDS group. As the original study was designed to investigate the influence of V_T_ and RR on pulse pressure variations, three variants of ventilation were used successively during each period: V1 = V_T_ 10 ml.kg^−1^ and RR 15 min^−1^; V2 = V_T_ 6 ml.kg^−1^ and RR 25 min^−1^; and V3 = V_T_ 6 ml.kg^−1^ and RR 15 min^−1^ (5–7 min each).

### Statistical analysis

Data are presented as mean ± SD or as mean changes between periods ± SE. Baseline measures (pre-ARDS) in the ARDS group were not included in the statistical analyses. To determine the influence of hemorrhage on measured variables, we used a mixed effects analysis of variance (ANOVA) with fixed effect for the 3 periods of the experiment and random effect for pig (8 or 10). The same model was used to analyze the changes of the major determinants of DO_2_ (CaO_2_, Hb and SaO_2_,) in the ARDS group only. This statistical model estimates all comparisons between time periods simultaneously, reducing the risk of type I error. Furthermore, it takes into account repeated measurements of each pig across periods and across variants of ventilation. We determined the significance of the effect of hemorrhage and retransfusion on these parameters. As the goal of the analysis was to determine the effect of acute hemorrhage on shunt and Crs and because in the CTRL group venous admixture rather than true shunt was estimated, we did not compare the two groups of animals. Results are presented as the mean difference between two periods (hemorrhage vs pre-hemorrhage; retransfusion vs hemorrhage; retransfusion vs prehemorrhage) with respective *p* values. Because SvO_2_ may influence PaO_2_:FiO_2_ through venous admixture [20] we also analyzed PaO_2_:FiO_2_ adjusted for SvO_2_. After visual inspection of the data in order to determine the effect of successive ventilation mode on shunt in the ARDS group, we performed a mixed effects analysis of variance of the shunt across variant of ventilation. Comparison of blood loss between the CTRL group and the ARDS group were performed using unpaired *t*-test. All analyses were performed with R software (Version 2.15.1). A value of *p* less than 0.05 was considered significant and all statistical tests were two-tailed.

## Results

**Fig. 1:**
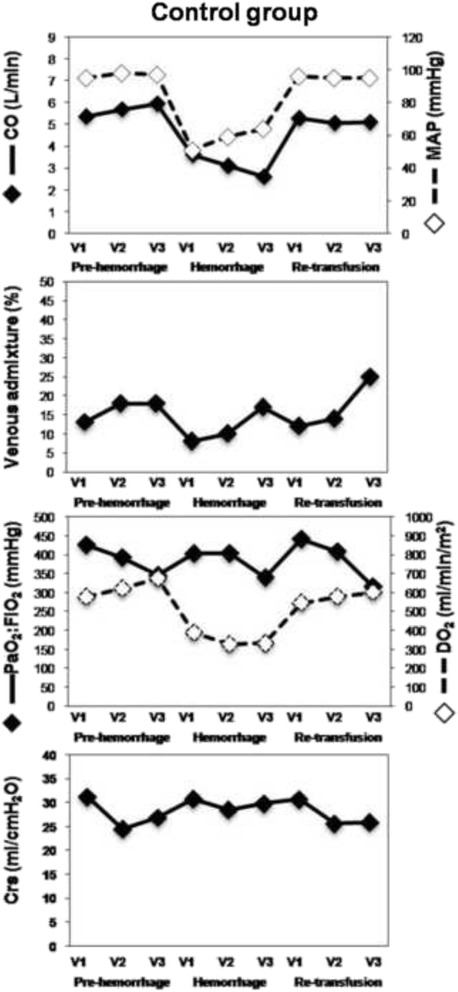
This figure shows the evolution of measured parameters during the experiment in the control group. All values are presented for each mode of ventilation (V1, V2, V3) in the successive phase of the experiment (Baseline, Hemorrhage, Re-transfusion). Crs, respiratory system compliance; CO, cardiac output; DO2, oxygen delivery; MAP, mean arterial pressure V1, tidal volume: 10 ml.kg^−1^ and respiratory rate: 15 min^−1^; V2 = tidal volume: 6 ml.kg^−1^ and respiratory rate: 25 min^−1^; V3, tidal volume 6 ml.kg^−1^ and respiratory rate: 15 min^−1^

**Fig. 2:**
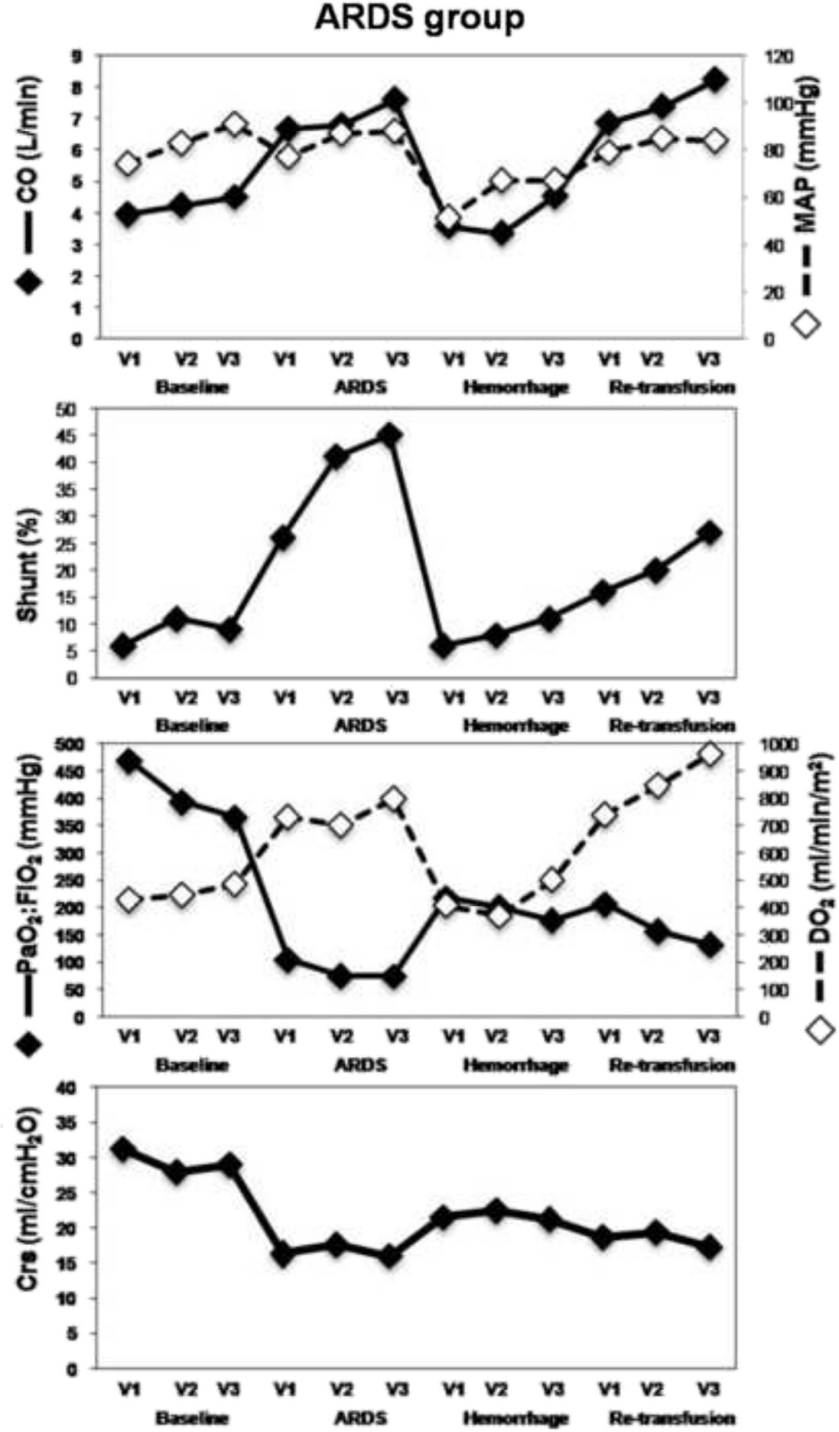
This figure shows the evolution of measured parameters during the experiment in the ARDS group. All values are presented for each mode of ventilation (V1, V2, V3) in the successive phase of the experiment (Baseline, ARDS, Hemorrhage, Re-transfusion). Crs, respiratory system compliance; CO, cardiac output; DO2, oxygen delivery; MAP, mean arterial pressure; shunt, intrapulmonary shunt; V1, tidal volume: 10 ml.kg^−1^ and respiratory rate: 15 min^−1^; V2 = tidal volume: 6 ml.kg^−1^ and respiratory rate: 25 min^−1^; V3, tidal volume 6 ml.kg^−1^ and respiratory rate: 15 min^−1^

Results are presented in Figs. 1 and 2, Tables 1 and 2. Mean difference and results of statistical analysis between periods are shown in Table 3.

**Table 1:** Hemodynamic, respiratory and oxygenation parameters during each mode of ventilation at each time point in the control group

	Pre-hemorrhage Mean ± SD				Hemorrhage Mean ± SD				Retransfusion Mean ± SD			
Ventilation mode:												
Tidal volume:ml.k g-1	VT:10	VT:6	VT:6	Average across variants of ventilation	VT:10	VT:6	VT:6	Average across variants of ventilation	VT:10	VT:6	VT:6	Average across variants of ventilation
Respiratory rate: breaths/min	RR: 15	RR: 25	RR: 15		RR: 15	RR: 25	RR: 15		RR: 15	RR: 25	RR: 15	
Cardiac output (l.min-1)	5.4 ± 1.4	5.7 ± 1.5	6.0 ± 1.9	5.7 ± 1.6	3.6 ± 0.8	3.1 ± 1.1	2.6 ± 1.6	3.1 ± 1	5.3 ± 1.7	5.1 ± 2.5	5.1 ± 2.6	5.1 ± 2.2
Heart Rate (bpm)	101 ± 32	100 ± 26	103 ± 24	101 ± 27	130 ± 25	154 ± 50	163 ± 55	150 ± 46	111 ± 30	114 ± 25	120 ± 27	115 ± 26
Mean arterial pressure (mmHg)	95 ± 13	98 ± 15	97 ± 14	96.8 ± 13.7	51 ± 15	59 ± 16	64 ± 13	58.1 ± 15.4	96 ± 10	95 ± 9	95 ± 10	95.2 ± 9.3
Central venous pressure (mmHg)	8 ± 1	7 ± 1	7 ± 2	8 ± 1	5 ± 2	5 ± 3	6 ± 3	5 ± 3	9 ± 2	7 ± 3	8 ± 1	8 ± 2
Veinous admixture (%)	13 ± 4	18 ± 8	18 ± 9	16 ± 7	8 ± 4	10 ± 4	17 ± 14	11 ± 9	12 ± 4	14 ± 5	25 ± 12	16 ± 9
PaO2:FiO2 (mmHg)	426 ± 72	393 ± 92	345 ± 91	388 ± 89	403 ± 72	404 ± 87	340 ± 90	385 ± 84	441 ± 59	408 ± 54	316 ± 82	395 ± 79
SVO2 (%)	78 ± 8	78 ± 5	76 ± 8	77 ± 7.0	60 ± 12	65 ± 10	64 ± 15	63 ± 12	82 ± 5	78 ± 8	77 ± 6	79 ± 7
Global oxygen delivery (ml.min-1.m-2)	575 ± 160	620 ± 201	674 ± 305	623 ± 225	388 ± 99	326 ± 147	333 ± 159	347 ± 132	544 ± 228	577 ± 205	601 ± 258	570 ± 216
Respiratory system compliance (ml.cm H_2_O^−1^)	31.2 ± 3.6	24.5 ± 5.1	26.9 ± 4.6	27.5 ± 5.1	30.8 ± 5.4	28.5 ± 2.9	29.8 ± 7.4	29.7 ± 5.2	30.7 ± 4.1	25.6 ± 4.2	25.9 ± 4.3	27.3 ± 4.6

**Table 2:** Hemodynamic, respiratory and oxygenation parameters during each mode of ventilation at each time point in the ARDS group (*n* = 8)

Ventilation mode	Pre-hemorrhage mean ± SD				Hemorrhage mean ± SD				Retransfusion mean ± SD			
Ventilation mode:												
VT:ml.kg-1	VT:10	VT:6	VT:6	Average across variants of ventilation	VT:10	VT:6	VT:6	Average across variants of ventilation	VT:10	VT:6	VT:6	Average across variants of ventilation
RR: breaths/min	RR: 15	RR: 25	RR: 15		RR: 15	RR: 25	RR: 15		RR: 15	RR: 25	RR: 15	
Cardiac output (l.min-1)	6.7 ± 1.3	6.8 ± 1.5	7.6 ± 1.4	7.0 ± 1.4	3.6 ± 0.9	3.4 ± 0.8	4.5 ± 1.0	3.8 ± 1.0	6.9 ± 3.0	7.4 ± 2.6	8.2 ± 2.1	7.5 ± 2.5
Heart rate (bpm)	145 ± 18	157 ± 30	171 ± 36	158 ± 30	210 ± 24	215 ± 17	209 ± 29	212 ± 22	172 ± 32	170 ± 27	181 ± 24	174 ± 27
Mean arterial pressure (mmHg)	77 ± 19	87 ± 14	88 ± 13	84.0 ± 16.0	51 ± 13	67 ± 16	67 ± 12	62 ± 15	79 ± 10	85 ± 10	84 ± 12	83 ± 11
Central venous pressure (mmHg)	8 ± 2	7 ± 2	7 ± 2	8 ± 2	6 ± 2	5 ± 1	5 ± 2	5 ± 1	9 ± 1	8 ± 1	8 ± 1	9 ± 1
Shunt (%)	26 ± 15	41 ± 12	45 ± 15	37 ± 16	6 ± 7	8 ± 6	11 ± 9	9 ± 7	16 ± 17	20 ± 16	27 ± 19	20 ± 18
PaO2:FiO2 (mmHg)	105 ± 38	76 ± 20	75 ± 19	85 ± 30	218 ± 105	201 ± 95	177 ± 80	199 ± 91	207 ± 125	157 ± 77	133 ± 59	166 ± 93
SVO2 (%)	69 ± 11	70 ± 9	69 ± 11	69 ± 10	59 ± 14	67 ± 15	67 ± 17	64 ± 15	79 ± 7	79 ± 8	81 ± 8	80 ± 7
Global oxygen delivery (ml.min-1.m-2)	731 ± 146	701 ± 219	798 ± 265	744 ± 210	408 ± 113	369 ± 114	501 ± 160	427 ± 137	740 ± 392	846 ± 404	961 ± 323	849 ± 370
Arterial content in oxygen (ml.dl-1)	11.0 ± 0.9	10.2 ± 1.5	10.4 ± 1.7	10.5 ± 1.4	10.9 ± 1.3	10.9 ± 1.5	11.0 ± 1.5	11.0 ± 1.4	11.5 ± 1.0	11.2 ± 1.6	11.6 ± 1.1	11.4 ± 1.2
Hemoglobin (g.dl-1)	8.4 ± 0.4	8.4 ± 0.6	8.6 ± 0.8	8.5 ± 0.6	7.6 ± 0.6	7.7 ± 0.8	7.9 ± 0.7	7.8 ± 0.7	8.1 ± 1.0	8.1 ± 1.3	8.6 ± 0.8	8.3 ± 1.0
SaO2 (%)	92 ± 7	86 ± 8	85 ± 8	87 ± 8	97 ± 4	96 ± 4	95 ± 6	96 ± 5	97 ± 4	95 ± 4	94 ± 6	95 ± 5
Respiratory sytem compliance (ml.cm H_2_O^−1^)	16.4 ± 3.0	17.6 ± 3.9	16.0 ± 2.6	16.8 ± 3.2	21.5 ± 2.9	22.5 ± 4.6	21.2 ± 4.3	21.9 ± 4	18.7 ± 2.7	19.4 ± 5.2	17.3 ± 2.9	18.5 ± 3.9

**Table 3:** Impact of hemorrhage and retransfusion on hemodynamic, respiratory and oxygenation parameters

Phase		Hemorrhage vs. pre-hemorrhage		Retransfusion vs. hemorrhage		Retransfusion vs. pre-hemorrhage	
Parameter	Group	Mean difference ± SE	*p*	Mean difference ± SE	*p*	Mean difference ± SE	*p*
*Hemodynamic*							
Cardiac output, l.min^−1^	CTRL	−2.6 ± 0.3	*<0.001*	2.4 ± 0.3	*<0.001*	−0.2 ± 0.3	*0.48*
	ARDS	−3.2 ± 0.4	*<0.001*	3.7 ± 0.4	*<0.001*	0.5 ± 0.4	*0.21*
Mean arterial pressure, mmHg	CTRL	−36.7 ± 3.1	*<0.001*	36.6 ± 3.2	*<0.001*	−0.1 ± 3.1	*0.98*
	ARDS	−22 .4 ± 4.0	*<0.001*	21.4 ± 4.1	*<0.001*	−1.0 ± 4.1	*0.80*
Central venous pressure, mmHg	CTRL	−2.3 ± 0.3	*<0.001*	2.9 ± 0.4	*<0.001*	0.6 ± 0.3	*0.09*
	ARDS	−2.2 ± 0.3	*<0.001*	3.2 ± 0.3	*<0.001*	1 ± 0.3	*0.003*
*Respiratory system and oxygenation*							
Venous admixture, %	CTRL	−6.1 ± 1.9	*0.002*	4.9 ± 1.9	*0.01*	−1.2 ± 1.8	*0.50*
Shunt, %	ARDS	−28.5 ± 3.5	*<0.001*	12.3 ± 3.5	*<0.001*	−16.1 ± 3.5	*<0.001*
PaO_2_:FiO_2_, mmHg	CTRL	1.7 ± 17.0	*0.92*	13.2 ± 17.2	*0.45*	14.5 ± 16.5	*0.37*
	ARDS	113.4 ± 19.1	*<0.001*	−33.3 ± 19.1	*0.09*	80.2 ± 19.1	*<0.001*
PaO_2_:FiO_2_ adjusted for mixed venous blood saturation, mmHg	CTRL	42.6 ± 23.8	*0.08*	−33.1 ± 25.3	*0.20*	9.4 ± 16.2	*0.56*
	ARDS	125.7 ± 19.2	*<0.001*	−71.3 ± 22.5	*0.002*	54.4 ± 20.6	*0.01*
Global oxygen delivery, ml.min-1.m^−2^	CTRL	−290.8 ± 37.0	*<0.001*	229.6 ± 38.4	*<0.001*	−61.1 ± 35.9	*0.09*
	ARDS	−316.9 ± 49.8	*<0.001*	422.1 ± 49.8	*<0.001*	105.3 ± 49.8	*0.04*
Arterial content in oxygen, ml.dl^−1^	ARDS	0.4 ± 0.6	*0.51*	0.5 ± 0.6	*0.48*	0.9 ± 0.6	*0.18*
Hemoglobin, g.dl^−1^	ARDS	−0.7 ± 0.4	*0.08*	0.5 ± 0.4	*0.18*	0.2 ± 0.4	*0.65*
Arterial blood saturation, %	ARDS	9.0 ± 2.6	*0.004*	−1.0 ± 2.6	*0.76*	8.0 ± 2.6	*0.008*
Respiratory system compliance, ml.cm H_2_O^−1^	CTRL	2.5 ± 0.9	*0.006*	−2.6 ± 0.9	*0.01*	−0.1 ± 0.9	*0.94*
	ARDS	5.1 ± 1.0	*<0.001*	−3.3 ± 1.0	*0.001*	1.8 ± 1.0	*0.07*

### Hemodynamics

The blood volume removed from the animals was 936 ± 100 ml (30 ml/kg) and 909 ± 94 ml (29 ml/kg) in the CTRL and ARDS group respectively (*p* > 0.05). CO (Figs. 1 and 2), mean arterial pressure and CVP decreased during the hemorrhage and recovered to baseline values during retransfusion (Table 3). The only exception was CVP in the ARDS group, which was slightly but significantly higher after retransfusion than pre-hemorrhage.

### Gas exchange, oxygen delivery and compliance

In the CTRL group (Fig. 1), venous admixture decreased during hemorrhage and recovered to pre-hemorrhage values during retransfusion (Table 1). In the ARDS group (Fig. 2), shunt decreased during hemorrhage and recovered during re-transfusion, but did not fully return to pre-hemorrhage values (Table 2). In the CTRL group (Fig. 1), even if shunt changed, PaO_2_:FiO_2_, was similar across pre-hemorrhage, hemorrhage and retransfusion phases (Table 1). On the other hand, in the ARDS group (Fig. 2), PaO_2_:FiO_2_ varies inversely from shunt: increased during hemorrhage and did not recover completely during retransfusion (Table 2). The adjustment of PaO_2_:FiO_2_ for SvO_2_ did not change these results.

For both groups, DO_2_ varied in accordance with CO. That is, DO_2_ decreased during hemorrhage and then recovered during retransfusion (CTRL group: Fig. 1; Table 1; ARDS group: Fig. 2; Table 2). The main determinants of DO_2_ were examined in the ARDS group. CaO_2_ did not change across the phases (Table 2), Hb remained stable across the phases and SaO_2_ increased during hemorrhage but did not recover during re-transfusion (Table 2).

For both groups, Crs increased during hemorrhage and recovered to pre-hemorrhage values during retransfusion (CTRL group: Fig. 1; Table 1; ARDS group: Fig. 2; Table 2).

### Impact of changes in ventilation settings on shunt in the ARDS group

At baseline (pre-ARDS), shunt did not change with ventilation setting (V1 to V2 change = 5.12 % (*p* = 0.19), V2 to V3 change = −1.83 (*p* = 0.61)). After ARDS induction but before hemorrhage (pre-hemorrhage period), shunt was only affected by the change from V1 to V2 (change = 15.16, *p* = 0.01) but not from V2 to V3 (change = 3.10, *p* = 0.57). After hemorrhage and retransfusion, there were no statistically significant differences in shunt values when ventilation was switched from V1 to V2 or from V2 to V3.

## Discussion

In the present animal study, the authors investigated the effect of a decrease in CO through acute severe hypovolemia (with neither a pharmacologic intervention nor a mechanical procedure) on intra-pulmonary shunt in healthy and ARDS animals. Our analysis suggests that severe hypovolemia induced by acute hemorrhage is associated with a decrease in shunt and an increase in Crs in a pig model of ARDS. These changes were associated with improvements in PaO_2_:FiO_2_ only at high values of shunt (ARDS-like syndrome). The improvement in PaO_2_:FiO_2_ did not compensate for the decrease in CO and, as a consequence, DO_2_ decreased. To our knowledge, this is the first study showing the effects of acute hemorrhage on Crs in ARDS pig model.

In the present study, we observed that shunt and blood oxygenation were influenced by acute hemorrhage (shunt decrease) and retransfusion (shunt increase). Moreover, as during ARDS oxygenation is closely related to shunt [21, 22], PaO_2_:FiO_2_ varied similarly to shunt. This relation between global perfusion and shunt has already been reported during a decrease and an increase in perfusion [4, 6–10]. However, the present study extends these results to demonstrate that this relation is associated with concomitant changes in respiratory system compliance and that the effects on PaO_2_:FiO_2_ are not observed for relatively moderate values of shunt.

**Fig. 3:**
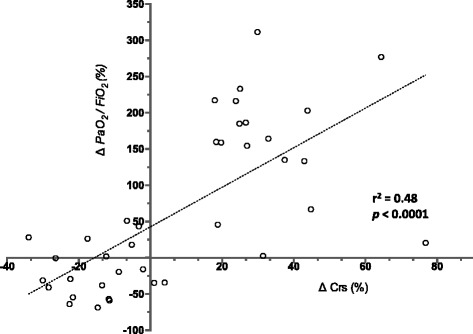
This figure report the relation between the changes (%) in PaO_2_:FiO_2_ and the changes (%) in Crs after hemorrhage and retransfusion. The correlation coefficient (r^2^) describes how two variables vary together

The decrease in shunt during hemorrhage could be explained by several physiological mechanisms. Firstly, it may be related to a preferential decrease in the perfusion of low- and/or non-ventilated regions. Indeed, to have a significant impact on oxygenation, the decrease in lung perfusion must be larger in the shunted part of the lung than in other normal lung regions. In ARDS, shunt essentially resides in the injured areas of the lung, in which perfusion is already limited by microvascular obstruction [23], adaptative hypoxic vasoconstriction [24], and gravitational compression of vessels [25, 26]. Secondly, in regions with lung injury, the impaired oxygenation and apparent “shunt” could also be explained by a decrease in pulmonary transit time in which the blood cannot be fully oxygenated because of increased red cell velocity [27, 28]. Thus, when the animals are hemorrhaged and pulmonary flow reduced, the reduced flow velocity may be coupled to a more efficient blood oxygenation. Finally, changes in Crs may also have influenced PaO_2_:FiO_2_ as suggested by the significant correlation between the changes in Crs and the changes in PaO_2_:FiO_2_ observed in our analysis and reported in Fig. 3. As the increase in Crs decreased alveolar pressures it may result in an improvement in the perfusion of West zone II [29] that may have reduced shunt.

The other means by which shunt could be decreased is to recruit poorly or non-ventilated lung. However, in our analysis, the decrease in shunt and the concurrent improvement in PaO_2_:FiO_2_ remained significant whatever the V_T_ value. This may be related to the fact that, during ARDS, pure shunt is predominant [6, 10] and small changes in V_T_ value will not have a significant impact. Indeed, in the ARDS group, shunt significantly increased when V_T_ was decreased from 10 ml.kg^−1^ (V1) to 6 ml.kg^−1^ (V2) during normovolemia but not during hemorrhage, confirming that the circulatory state was the main determinant of the shunt during Hypovolemia (Fig. 1; Table 2). As well, although the calculated shunt in the CTRL group was overestimated (since the calculation of shunt at FiO_2_ lower than 100 % represents venous admixture and not a true shunt [30]), the impact of bleeding on CTRL group PaO_2_:FiO_2_ was low, demonstrating that the circulatory state remains a determinant of the shunt only when lungs are affected by atelectasis.

However, even if gas exchange (PaO_2_:FiO_2_) and SaO_2_ improved during hemorrhage, it does not result in better tissue oxygenation. Indeed, the improvement in SaO_2_ was not large enough to influence CaO_2_, due in part to a slight decrease in hemoglobin. As a consequence, the decrease in CO reduced DO_2_. However, as the initial study was not designed to measure tissue oxygenation abnormalities, the impact of this decrease of DO_2_ on other vital organs function have not be investigated in the present analysis. Nevertheless, our observation is relevant to clinical settings, as it indicates that an improvement in PaO_2_:FiO_2_ and SaO_2_ can falsely mask a worsening in systemic oxygen delivery.

A key result in this study is the observed changes in Crs following blood volume shift (hemorrhage and retransfusion) (Table 3). An influence of blood volume on Crs has already been reported during blood volume decrease [12, 13] or increase [16], but previous studies reported conflicting results as they reported either a decrease [5, 31] or a stable [14, 15] Crs following hemorrhage. None of these studies investigated this effect during ARDS. One explanation for the inconsistency in previous results could be related to the variable and interrelated impact of blood volume on the total lung volume (including air, blood and tissue) and the participation of the microvascular network to the stiffness of the lung tissue. Indeed, considering the relation between pulmonary vascular pressure/filling and lung mechanical characteristics, various studies suggests that the pressurized pulmonary capillary network act as a tether for the lung tissue which may influence respiratory mechanics stability [32, 33].

This study acknowledges some limitations. First of all, the present report and analysis are based on previous experimentations [17] in which the main goal was far away from considering the impact of CO on intrapulmonary shunt. However, this secondary analysis of previously collected data is an ethical way to study further concepts without performing new experimentations and minimizing, by the way, the use of animals. Moreover, even if the design of the initial study was not specifically adapted to the aim of the present analysis, our results are in accordance with physiological laws and support previous works on this subject. Second, animals were ventilated without PEEP. Adding PEEP could have limited the development of atelectatic regions resulting in a model without significant shunt and decreased Crs, which could not allow investigating the specific effect of hemorrhage on these parameters. Consequently, we could not totally extrapolate the effects of hemorrhage on shunt and Crs in situations of ARDS ventilated with PEEP. However in patients with ARDS, even if high PEEP is used, the remaining hypoxemia is predominantly related to persistent shunt that could be influenced by the changes in CO. Third, our model of ARDS induced by a surfactant depletion method is a classical model of ARDS-like syndrome in animals resulting in reproducible and stable changes in physiological parameters similar to human ARDS [34]. However, this model is mainly characterized by atelectasis and less inflammatory lesions. Third, due to the design of the initial study [17], animals were ventilated with three successive ventilation modes. These changes in ventilatory settings could have influenced measured parameters. However, we used a mixed model ANOVA taking into account the effect of ventilation mode. Moreover, when we analyzed the impact of changes in ventilatory settings on shunt in the ARDS group, the mode of ventilation affected shunt only in the pre-hemorrhage period and between V1:V_T_: 10 ml.kg^−1^, RR: 15 min^−1^ and V2: V_T_: 6 ml.kg-1, RR: 25 min^−1^ but not in other period nor other change in ventilation mode. Therefore, the ventilation mode used in this study does not seem to influence significantly the measured parameters. Fourth, the translation of this physiological model in a clinical perspective is not straightforward: our model of acute and severe hemorrhage is not strictly equivalent to a progressive hypovolemia, as could be obtained by diuretic for example. However, our results consolidate the hypothesis that decrease in blood volume may influence respiratory system characteristics, especially when these characteristics are already altered. Moreover it emphasizes that the influence of a decrease in blood volume on SaO_2_ or PaO_2_:FiO_2_ may be at the expense of global oxygen delivery. Our model may be more closely encountered for example during the institution or the weaning of extracorporeal membrane oxygenation (ECMO) where large and acute changes in circulating blood volume may influence respiratory system characteristics. Finally, the compensatory release of catecholamine associated with the acute severe hemorrhage model may have influenced our observation. Indeed, catecholamine increase pulmonary vascular resistance [35] which may have amplified the decrease in pulmonary shunt observed during hemorrhage.

## Conclusion

Our observations show that there is an impact of blood volume on respiratory system characteristics and oxygenation parameters during ARDS. Indeed, we observed that an acute hemorrhage results in a decrease in shunt with a parallel improvement in arterial oxygenation. Moreover, after hemorrhage, Crs improves. Our observations illustrate the link between physiologic mechanisms and clinical observations and may explain why in clinical practice a rapid modest improvement in lung compliance and/or a large improvement in oxygenation parameters in patients with ARDS may not necessarily reflect an improvement in patient condition but rather may be related to an acute decrease in transpulmonary blood flow. Our analysis thus strengthens the importance to integrate the circulatory condition in the analysis of the state of the respiratory system. However, the caveats of the study (use of a model not replicable in clinical activity; lack of data on the impact of oxygen delivery on clinical outcome) do not allow having a reliable take-home message for the clinical management of ARDS.

### Ethics approval

The study was approved by the Ethics Committee for Animal Research at the University Medical Centre and by the Cantonal Veterinary Office of Geneva, Switzerland (No 31.1.1043/3127/1). Animals received respectful care in accordance with the Guide for the Care and Use of Laboratory Animals (Institute of Laboratory Animal Resources, 1996).

### Consent for publication

“Not applicable”.

### Availability of data and material

The data supporting our results are presented within the article.

### Availability of data and materials

Data are available upon request.

## Abbreviations

ABG: arterial blood gas
ARDS: acute respiratory distress syndrome
CaO_2_: arterial oxygen content
CcO_2_: pulmonary capillary oxygen content
CO: cardiac output
Crs: respiratory system compliance
CTRL group: no intervention
CvO_2_: mixed venous oxygen content
CVP: central venous pressure
DO_2_: global oxygen delivery
Hb: hemoglobin
PaO_2_:FiO_2_: arterial oxygenation
PEEP: positive end-expiratory pressure
Qs: shunt flow
Qs/Qt: Intrapulmonary shunt
Qt: represents the systemic blood flow
RR: respiratory rate
SaO_2_: oxygen saturation (arterial blood oxygen saturation
SvO_2_: mixed venous blood oxygen saturation
V_T_: tidal volume
